# The genome sequence of the Celtic sea slug, *Onchidella celtica* (Audouin & Milne-Edwards, 1832) (Systellommatophora: Onchidiidae)

**DOI:** 10.12688/wellcomeopenres.25000.1

**Published:** 2025-10-06

**Authors:** Nova Mieszkowska, Marine Biological Association Genome Acquisition Lab, Darwin Tree of Life Barcoding Collective, Wellcome Sanger Institute Tree of Life Management, Samples and Laboratory team, Wellcome Sanger Institute Scientific Operations: Sequencing Operations, Wellcome Sanger Institute Tree of Life Core Informatics team, Tree of Life Core Informatics collective, Darwin Tree of Life Consortium

**Affiliations:** 1 The Marine Biological Association, Plymouth, England, UK

**Keywords:** Onchidella celtica; Celtic sea slug; genome sequence; chromosomal; Systellommatophora

## Abstract

We present a genome assembly from an individual *Onchidella celtica* (Celtic sea slug; Mollusca; Gastropoda; Systellommatophora; Onchidiidae). The genome sequence has a total length of 1 141.02 megabases. Most of the assembly (99.92%) is scaffolded into 18 chromosomal pseudomolecules. Gene annotation of this assembly on Ensembl identified 12 582 protein-coding genes. The mitochondrial genome has also been assembled, with a length of 13.99 kilobases. This assembly was generated as part of the Darwin Tree of Life project, which produces reference genomes for eukaryotic species found in Britain and Ireland.

## Species taxonomy

Eukaryota; Opisthokonta; Metazoa; Eumetazoa; Bilateria; Protostomia; Spiralia; Lophotrochozoa; Mollusca; Gastropoda; Heterobranchia; Euthyneura; Panpulmonata; Eupulmonata; Systellommatophora; Onchidioidea; Onchidiidae; *Onchidella*; *Onchidella celtica* (Audouin & Milne-Edwards, 1832) (NCBI:txid36933)

## Background

*Onchidella celtica* (Audouin & Milne-Edwards, 1832) is a small (up to 12 mm in length and 6 mm width) marine pulmonate gastropod mollusc from the family Onchidiidae. Its recorded distribution range extends from Mauritania to northwest Scotland, being predominantly found on the western coastlines of the UK ( *Onchidella celtica* distribution map. World Register of Marine Species, 2025; *Onchidella celtica*. NBN Atlas species page, 2025). It occurs on exposed rocky shores ( Forbes *et al.*, 1853; Jeffreys, 1867; Joyeux-Laffuie, 1882), living gregariously amongst mussels, barnacles, and in rock crevices ( Beauchamp, 1923; Fretter, 1943; Russell, 1925). It forages when the tide is out, feeding on juvenile algae and diatoms ( Fretter, 1943; Kent & Hawkins, 2019), and is more commonly observed in damp, humid conditions (Mieszkowska pers. obs), likely accounting for the observed locally patchy distribution ( Dron, 1971; Joyeux-Laffuie, 1882; Russell, 1925). Various reports of the vertical distribution of *O. celtica* exist within the literature ( Jeffreys, 1867), however, more recent work favours a vertical range coinciding with that of neap tides and possibly a little higher vertically than the high neap tide level ( Britton, 1982; Dron, 1971).

This air-breathing sea slug has an oval body with a mantle that is black to dark green in colour and is covered in large tubercles. There are two short, thick, cylindrical tentacles with eyes at the tips that are located on the head ( Rowley, 2005). As a pulmonate gastropod, it has no gills, with the mantle cavity acting as a lung ( Barfield, 2003). *O. celtica* is a hermaphrodite, laying from 60 to 100 eggs in tubular capsules approximately 1 cm in diameter, and undergoing reciprocal fertilisation ( Barfield, 2003).

**Figure 1.**
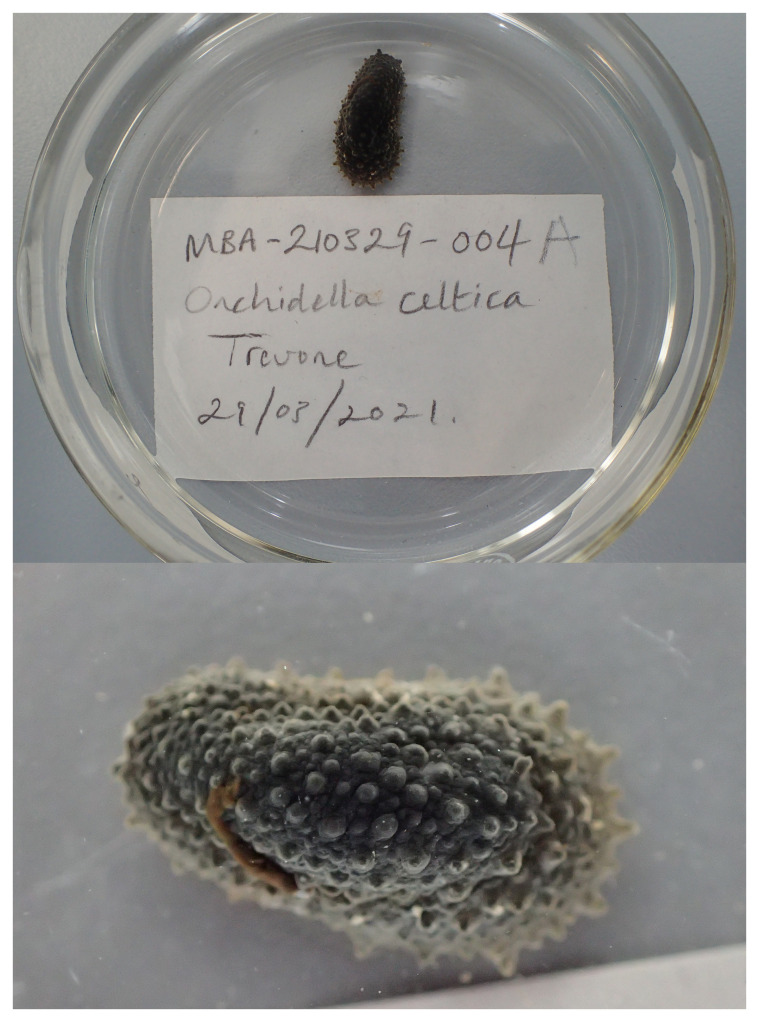
Photographs of the *Onchidella celtica* (xgOncCelt3) specimen used for genome sequencing.

We present a chromosome-level genome sequence for *Onchidella celtica*, the first high-quality genome for the genus *Onchidella* (data obtained via NCBI datasets, O’Leary *et al.*, 2024). The assembly was produced using the Tree of Life pipeline from a specimen collected in Trevone, Cornwall, United Kingdom ( Figure 1). This assembly was generated as part of the Darwin Tree of Life Project, which aims to generate high-quality reference genomes for all named eukaryotic species in Britain and Ireland to support research, conservation, and the sustainable use of biodiversity ( Blaxter *et al.*, 2022).

## Methods

### Sample acquisition and DNA barcoding

The specimen used for genome sequencing was an adult *Onchidella celtica* (specimen ID MBA-210329-004A, ToLID xgOncCelt3; Figure 1), collected from Trevone, Cornwall, United Kingdom (latitude 50.545, longitude –4.985) on 2021-03-29. The specimen was collected and identified by Nova Mieszkowska (Marine Biological Association). For the Darwin Tree of Life sampling and metadata approach, refer to Lawniczak *et al*. (2022).

The initial identification was verified by an additional DNA barcoding process according to the framework developed by Twyford *et al.* (2024). A small sample was dissected from the specimen and stored in ethanol, while the remaining parts were shipped on dry ice to the Wellcome Sanger Institute (WSI) (see the protocol). The tissue was lysed, the COI marker region was amplified by PCR, and amplicons were sequenced and compared to the BOLD database, confirming the species identification ( Crowley *et al.*, 2023). Following whole genome sequence generation, the relevant DNA barcode region was also used alongside the initial barcoding data for sample tracking at the WSI ( Twyford *et al.*, 2024). The standard operating procedures for Darwin Tree of Life barcoding are available on protocols.io.

### Nucleic acid extraction

Protocols for high molecular weight (HMW) DNA extraction developed at the Wellcome Sanger Institute (WSI) Tree of Life Core Laboratory are available on protocols.io ( Howard *et al.*, 2025). The xgOncCelt3 sample was weighed and triaged to determine the appropriate extraction protocol. Tissue from the anterior body was homogenised by powermashing using a PowerMasher II tissue disruptor. HMW DNA was extracted using the Automated MagAttract v2 protocol. DNA was sheared into an average fragment size of 12–20 kb following the Megaruptor®3 for LI PacBio protocol. Sheared DNA was purified by automated SPRI (solid-phase reversible immobilisation). The concentration of the sheared and purified DNA was assessed using a Nanodrop spectrophotometer and Qubit Fluorometer using the Qubit dsDNA High Sensitivity Assay kit. Fragment size distribution was evaluated by running the sample on the FemtoPulse system. For this sample, the final post-shearing DNA had a Qubit concentration of 29.2 ng/μL and a yield of 3 796.00 ng.

### PacBio HiFi library preparation and sequencing

Library preparation and sequencing were performed at the WSI Scientific Operations core. Libraries were prepared using the SMRTbell Prep Kit 3.0 (Pacific Biosciences, California, USA), following the manufacturer’s instructions. The kit includes reagents for end repair/A-tailing, adapter ligation, post-ligation SMRTbell bead clean-up, and nuclease treatment. Size selection and clean-up were performed using diluted AMPure PB beads (Pacific Biosciences). DNA concentration was quantified using a Qubit Fluorometer v4.0 (ThermoFisher Scientific) and the Qubit 1X dsDNA HS assay kit. Final library fragment size was assessed with the Agilent Femto Pulse Automated Pulsed Field CE Instrument (Agilent Technologies) using the gDNA 55 kb BAC analysis kit.

The sample was sequenced on a Revio instrument (Pacific Biosciences). The prepared library was normalised to 2 nM, and 15 μL was used for making complexes. Primers were annealed and polymerases bound to generate circularised complexes, following the manufacturer’s instructions. Complexes were purified using 1.2X SMRTbell beads, then diluted to the Revio loading concentration (200–300 pM) and spiked with a Revio sequencing internal control. The sample was sequenced on a Revio 25M SMRT cell. The SMRT Link software (Pacific Biosciences), a web-based workflow manager, was used to configure and monitor the run and to carry out primary and secondary data analysis.

### Hi-C

***Sample preparation and crosslinking***

The Hi-C sample was prepared from 20–50 mg of frozen anterior body tissue of the xgOncCelt3 sample using the Arima-HiC v2 kit (Arima Genomics). Following the manufacturer’s instructions, tissue was fixed and DNA crosslinked using TC buffer to a final formaldehyde concentration of 2%. The tissue was homogenised using the Diagnocine Power Masher-II. Crosslinked DNA was digested with a restriction enzyme master mix, biotinylated, and ligated. Clean-up was performed with SPRISelect beads before library preparation. DNA concentration was measured with the Qubit Fluorometer (Thermo Fisher Scientific) and Qubit HS Assay Kit. The biotinylation percentage was estimated using the Arima-HiC v2 QC beads.

***Hi-C library preparation and sequencing***

Biotinylated DNA constructs were fragmented using a Covaris E220 sonicator and size selected to 400–600 bp using SPRISelect beads. DNA was enriched with Arima-HiC v2 kit Enrichment beads. End repair, A-tailing, and adapter ligation were carried out with the NEBNext Ultra II DNA Library Prep Kit (New England Biolabs), following a modified protocol where library preparation occurs while DNA remains bound to the Enrichment beads. Library amplification was performed using KAPA HiFi HotStart mix and a custom Unique Dual Index (UDI) barcode set (Integrated DNA Technologies). Depending on sample concentration and biotinylation percentage determined at the crosslinking stage, libraries were amplified with 10–16 PCR cycles. Post-PCR clean-up was performed with SPRISelect beads. Libraries were quantified using the AccuClear Ultra High Sensitivity dsDNA Standards Assay Kit (Biotium) and a FLUOstar Omega plate reader (BMG Labtech).

Prior to sequencing, libraries were normalised to 10 ng/μL. Normalised libraries were quantified again and equimolar and/or weighted 2.8 nM pools. Pool concentrations were checked using the Agilent 4200 TapeStation (Agilent) with High Sensitivity D500 reagents before sequencing. Sequencing was performed using paired-end 150 bp reads on the Illumina NovaSeq 6000.

### Genome assembly

Prior to assembly of the PacBio HiFi reads, a database of *k*-mer counts ( *k* = 31) was generated from the filtered reads using FastK. GenomeScope2 ( Ranallo-Benavidez *et al.*, 2020) was used to analyse the *k*-mer frequency distributions, providing estimates of genome size, heterozygosity, and repeat content.

The HiFi reads were assembled using Hifiasm ( Cheng *et al.*, 2021) with the --primary option. Haplotypic duplications were identified and removed using purge_dups ( Guan *et al.*, 2020). The Hi-C reads ( Rao *et al.*, 2014) were mapped to the primary contigs using bwa-mem2 ( Vasimuddin *et al.*, 2019), and the contigs were scaffolded in YaHS ( Zhou *et al.*, 2023) with the --break option for handling potential misassemblies. The scaffolded assemblies were evaluated using Gfastats ( Formenti *et al.*, 2022), BUSCO ( Manni *et al.*, 2021) and MERQURY.FK ( Rhie *et al.*, 2020).

The mitochondrial genome was assembled using MitoHiFi ( Uliano-Silva *et al.*, 2023), which runs MitoFinder ( Allio *et al.*, 2020) and uses these annotations to select the final mitochondrial contig and to ensure the general quality of the sequence.

### Assembly curation

The assembly was decontaminated using the Assembly Screen for Cobionts and Contaminants ( ASCC) pipeline. TreeVal was used to generate the flat files and maps for use in curation. Manual curation was conducted primarily in PretextView and HiGlass ( Kerpedjiev *et al.*, 2018). Scaffolds were visually inspected and corrected as described by Howe *et al*. (2021). Manual corrections included 18 breaks, 35 joins, and removal of 1 haplotypic duplication. The curation process is documented at https://gitlab.com/wtsi-grit/rapid-curation. PretextSnapshot was used to generate a Hi-C contact map of the final assembly.

### Assembly quality assessment

The Merqury.FK tool ( Rhie *et al.*, 2020) was run in a Singularity container ( Kurtzer *et al.*, 2017) to evaluate *k*-mer completeness and assembly quality for the primary and alternate haplotypes using the *k*-mer databases ( *k* = 31) computed prior to genome assembly. The analysis outputs included assembly QV scores and completeness statistics.

The genome was analysed using the BlobToolKit pipeline, a Nextflow implementation of the earlier Snakemake version ( Challis *et al.*, 2020). The pipeline aligns PacBio reads using minimap2 ( Li, 2018) and SAMtools ( Danecek *et al.*, 2021) to generate coverage tracks. It runs BUSCO ( Manni *et al.*, 2021) using lineages identified from the NCBI Taxonomy ( Schoch *et al.*, 2020). For the three domain-level lineages, BUSCO genes are aligned to the UniProt Reference Proteomes database ( Bateman *et al.*, 2023) using DIAMOND blastp ( Buchfink *et al.*, 2021). The genome is divided into chunks based on the density of BUSCO genes from the closest taxonomic lineage, and each chunk is aligned to the UniProt Reference Proteomes database with DIAMOND blastx. Sequences without hits are chunked using seqtk and aligned to the NT database with blastn ( Altschul *et al.*, 1990). The BlobToolKit suite consolidates all outputs into a blobdir for visualisation. The BlobToolKit pipeline was developed using nf-core tooling ( Ewels *et al.*, 2020) and MultiQC ( Ewels *et al.*, 2016), with containerisation through Docker ( Merkel, 2014) and Singularity ( Kurtzer *et al.*, 2017).

## Genome sequence report

### Sequence data

**Figure 2.**
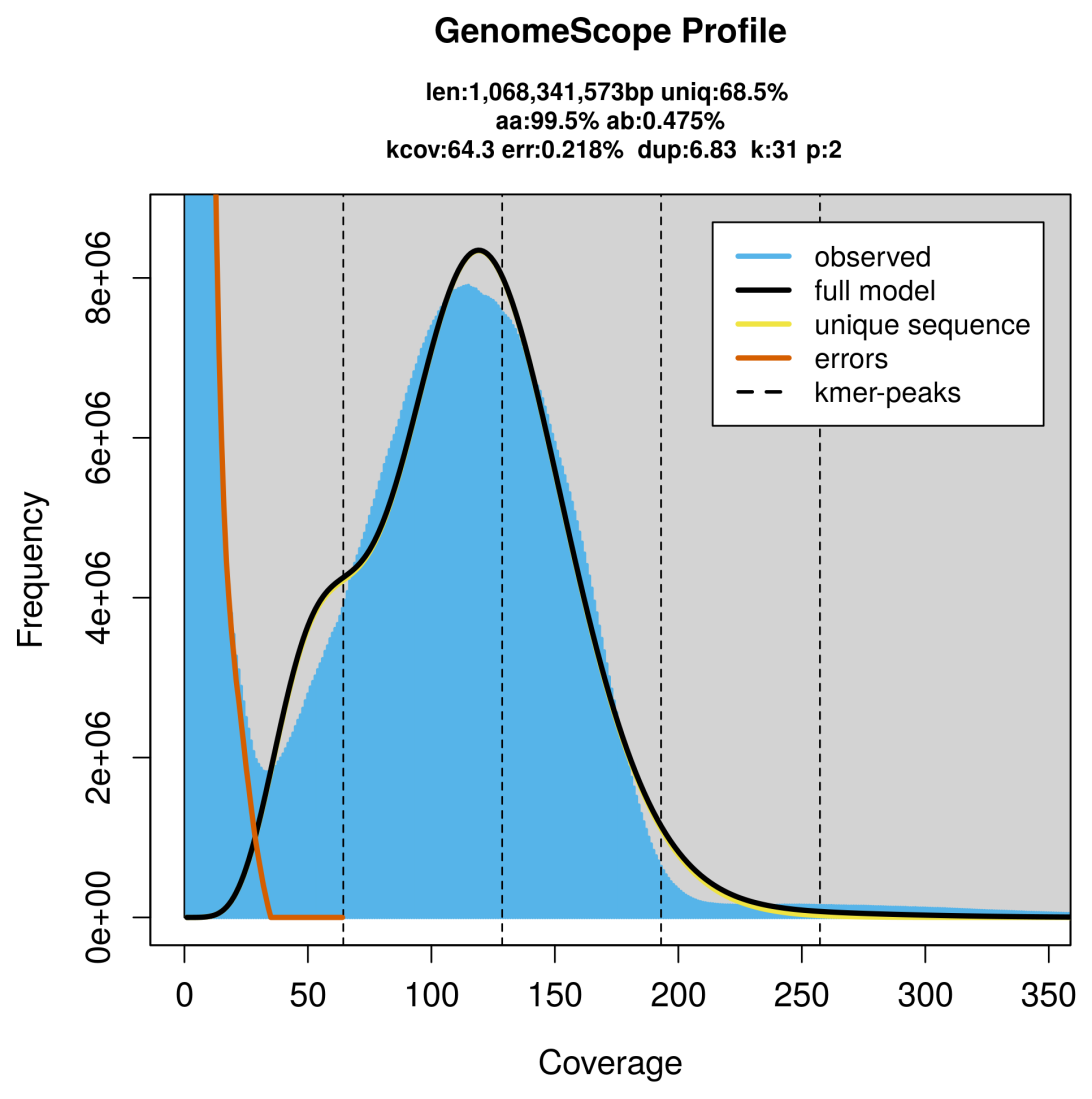
Frequency distribution of *k*-mers generated using GenomeScope2. The plot shows observed and modelled *k*-mer spectra, providing estimates of genome size, heterozygosity, and repeat content based on unassembled sequencing reads.

PacBio sequencing of the *Onchidella celtica* specimen generated 81.19 Gb (gigabases) from 9.30 million reads, which were used to assemble the genome. GenomeScope2.0 analysis estimated the haploid genome size at 1 068.34 Mb, with a heterozygosity of 0.47% and repeat content of 31.83% ( Figure 2). These estimates guided expectations for the assembly. Based on the estimated genome size, the sequencing data provided approximately 71× coverage. Hi-C sequencing produced 438.64 Gb from 2 904.90 million reads, which were used to scaffold the assembly. Table 1 summarises the specimen and sequencing details.

**Table 1.** Specimen and sequencing data for BioProject PRJEB71538.

Platform	PacBio HiFi	Hi-C
**ToLID**	xgOncCelt3	xgOncCelt3
**Specimen ID**	MBA-210329-004A	MBA-210329-004A
**BioSample (source individual)**	SAMEA110449713	SAMEA110449713
**BioSample (tissue)**	SAMEA110450538	SAMEA110450538
**Tissue**	anterior body	anterior body
**Instrument**	Revio	Illumina NovaSeq 6000
**Run accessions**	ERR12408789; ERR12408788	ERR12512733
**Read count total**	9.30 million	2 904.90 million
**Base count total**	81.19 Gb	438.64 Gb

### Assembly statistics

The primary haplotype was assembled, and contigs corresponding to an alternate haplotype were also deposited in INSDC databases. The final assembly has a total length of 1 141.02 Mb in 114 scaffolds, with 923 gaps, and a scaffold N50 of 61.4 Mb ( Table 2).

**Table 2.** Genome assembly statistics.

**Assembly name**	xgOncCelt3.1
**Assembly accession**	GCA_963931925.1
**Alternate haplotype accession**	GCA_963931835.1
**Assembly level**	chromosome
**Span (Mb)**	1 141.02
**Number of chromosomes**	18
**Number of contigs**	1 037
**Contig N50**	1.96 Mb
**Number of scaffolds**	114
**Scaffold N50**	61.4 Mb
**Organelles**	Mitochondrion: 13.99 kb

**Figure 3.**
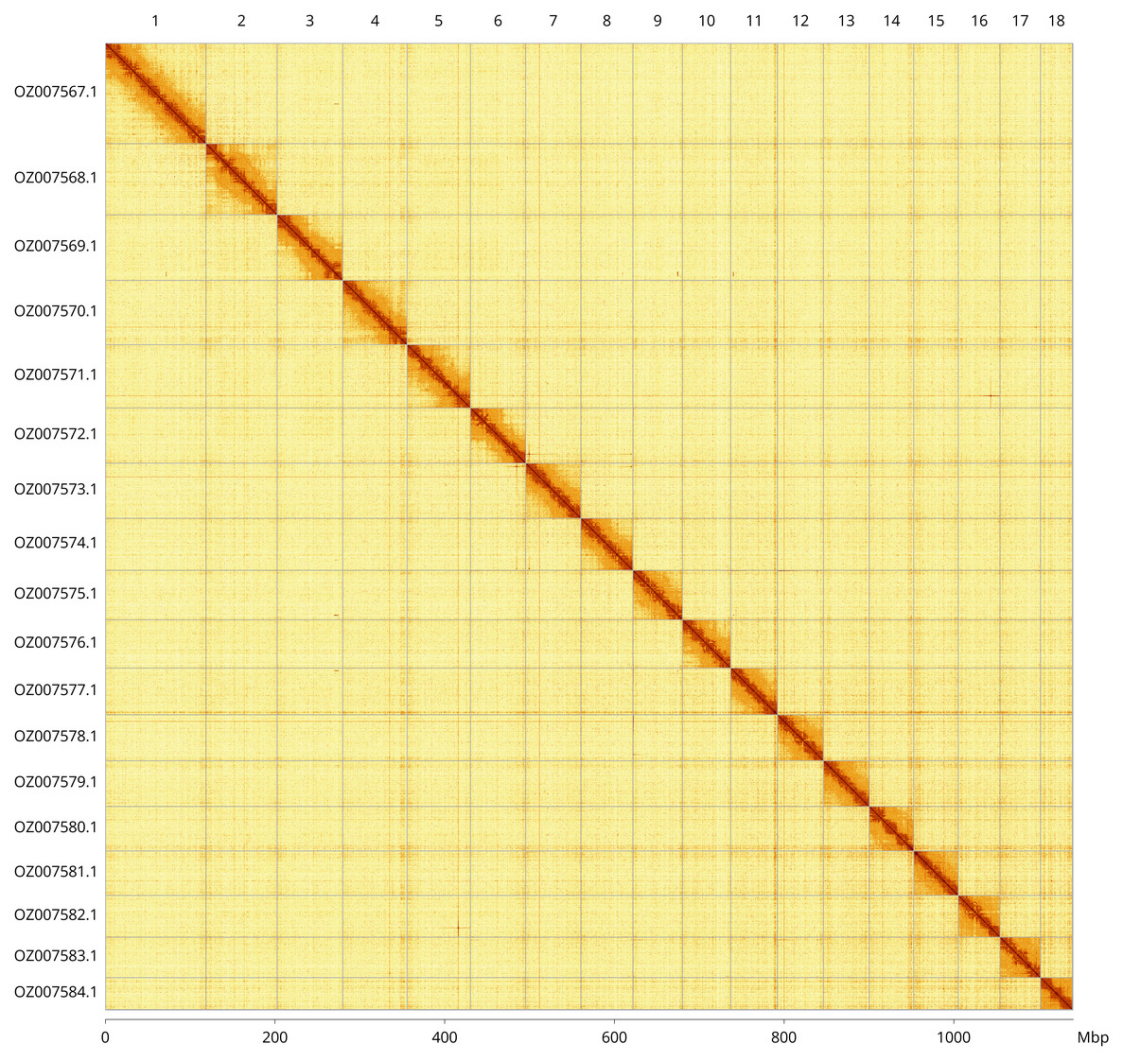
Hi-C contact map of the *Onchidella celtica* genome assembly. Assembled chromosomes are shown in order of size and labelled along the axes, with a megabase scale shown below. The plot was generated using PretextSnapshot.

Most of the assembly sequence (99.92%) was assigned to 18 chromosomal-level scaffolds. These chromosome-level scaffolds, confirmed by Hi-C data, are named according to size ( Figure 3; Table 3). The mitochondrial genome was also assembled. This sequence is included as a contig in the multifasta file of the genome submission and as a standalone record.

**Table 3.** Chromosomal pseudomolecules in the primary genome assembly of *Onchidella celtica* xgOncCelt3.

INSDC accession	Molecule	Length (Mb)	GC%
OZ007567.1	1	118.62	39.50
OZ007568.1	2	83.86	40
OZ007569.1	3	77.53	40
OZ007570.1	4	75.73	40
OZ007571.1	5	74.65	40
OZ007572.1	6	65.26	40
OZ007573.1	7	64.92	40.50
OZ007574.1	8	61.40	40
OZ007575.1	9	58.44	40
OZ007576.1	10	56.89	40.50
OZ007577.1	11	55.03	40.50
OZ007578.1	12	54.29	40.50
OZ007579.1	13	53.82	40.50
OZ007580.1	14	52.58	40.50
OZ007581.1	15	52.35	40.50
OZ007582.1	16	49.40	40.50
OZ007583.1	17	47.46	40.50
OZ007584.1	18	37.82	40.50

### Assembly quality metrics

**Figure 4.**
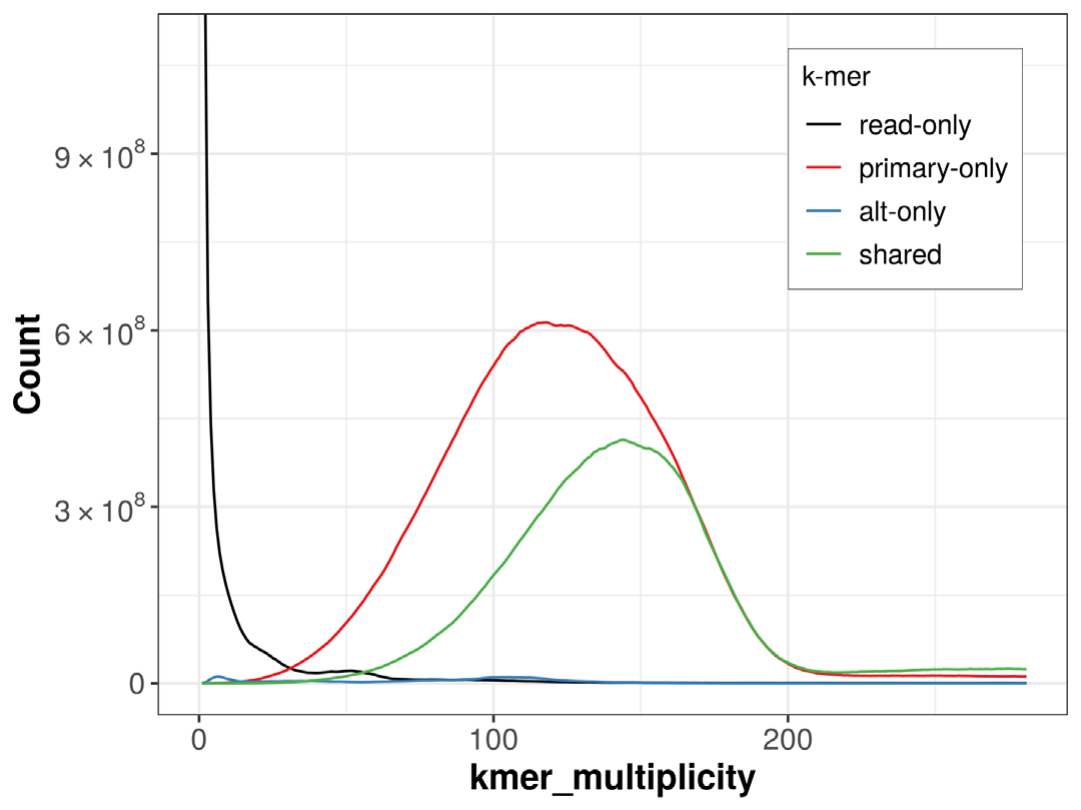
Evaluation of *k*-mer completeness using MerquryFK. This plot illustrates the recovery of *k*-mers from the original read data in the final assemblies. The horizontal axis represents *k*-mer multiplicity, and the vertical axis shows the number of *k*-mers. The black curve represents *k*-mers that appear in the reads but are not assembled. The green curve corresponds to *k*-mers shared by both haplotypes, and the red and blue curves show *k*-mers found only in one of the haplotypes.

The combined primary and alternate assemblies achieve an estimated QV of 56.0. The *k*-mer completeness is 97.06% for the primary assembly, 33.36% for the alternate haplotype, and 97.98% for the combined assemblies ( Figure 4).

**Figure 5.**
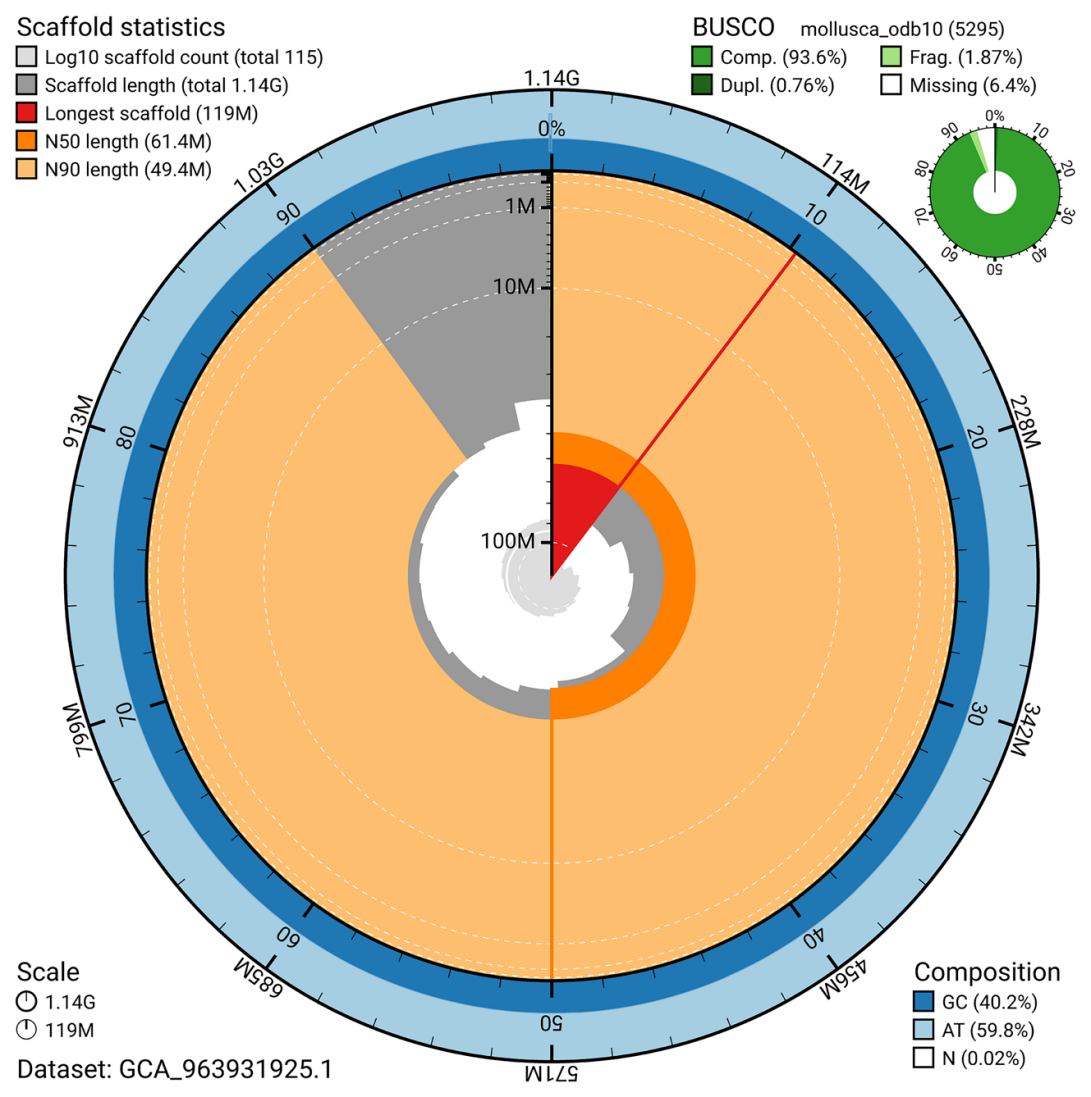
Assembly metrics for xgOncCelt3.1. The BlobToolKit snail plot provides an overview of assembly metrics and BUSCO gene completeness. The circumference represents the length of the whole genome sequence, and the main plot is divided into 1 000 bins around the circumference. The outermost blue tracks display the distribution of GC, AT, and N percentages across the bins. Scaffolds are arranged clockwise from longest to shortest and are depicted in dark grey. The longest scaffold is indicated by the red arc, and the deeper orange and pale orange arcs represent the N50 and N90 lengths. A light grey spiral at the centre shows the cumulative scaffold count on a logarithmic scale. A summary of complete, fragmented, duplicated, and missing BUSCO genes in the mollusca_odb10 set is presented at the top right. An interactive version of this figure can be accessed on the BlobToolKit viewer.

**Figure 6.**
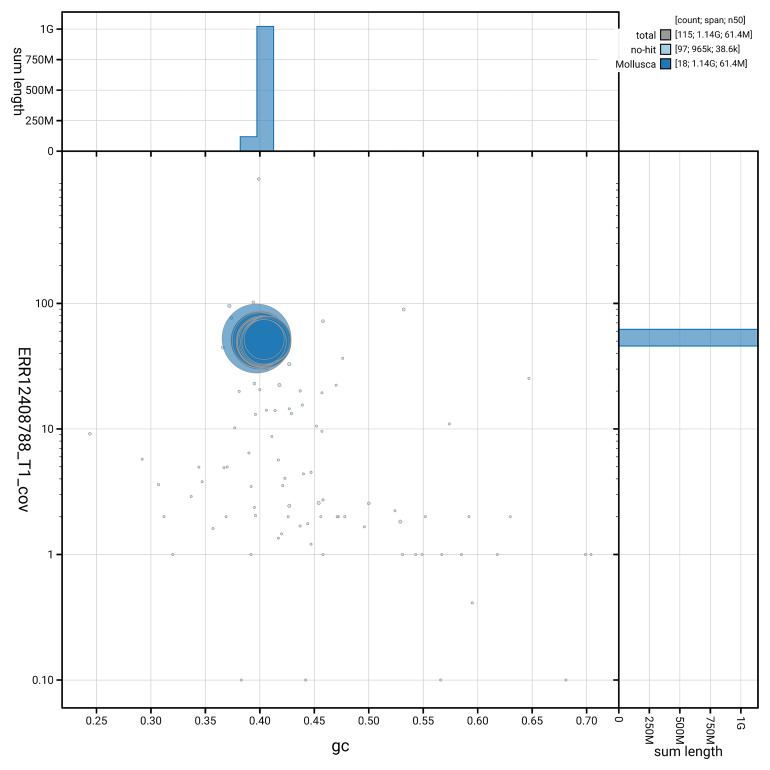
BlobToolKit GC-coverage plot for xgOncCelt3.1. Blob plot showing sequence coverage (vertical axis) and GC content (horizontal axis). The circles represent scaffolds, with the size proportional to scaffold length and the colour representing phylum membership. The histograms along the axes display the total length of sequences distributed across different levels of coverage and GC content. An interactive version of this figure is available on the BlobToolKit viewer.

BUSCO v.5.5.0 analysis using the mollusca_odb10 reference set ( *n* = 5 295) identified 93.6% of the expected gene set (single = 92.8%, duplicated = 0.8%). The snail plot in Figure 5 summarises the scaffold length distribution and other assembly statistics for the primary assembly. The blob plot in Figure 6 shows the distribution of scaffolds by GC proportion and coverage.

Table 4 lists the assembly metric benchmarks adapted from Rhie *et al.* (2021) the Earth BioGenome Project Report on Assembly Standards September 2024. The EBP metric, calculated for the primary assembly, is **6.C.Q56**, meeting the recommended reference standard.

**Table 4.** Earth Biogenome Project summary metrics for the *Onchidella celtica* assembly.

Measure	Value	Benchmark
EBP summary (primary)	6.C.Q56	6.C.Q40
Contig N50 length	1.96 Mb	≥ 1 Mb
Scaffold N50 length	61.40 Mb	= chromosome N50
Consensus quality (QV)	Primary: 56.8; alternate: 54.9; combined: 56.0	≥ 40
*k*-mer completeness	Primary: 97.06%; alternate: 33.36%; combined: 97.98%	≥ 95%
BUSCO	C:93.6% [S:92.8%; D:0.8%]; F:1.9%; M:4.5%; n:5 295	S > 90%; D < 5%
Percentage of assembly assigned to chromosomes	99.92%	≥ 90%

## Genome annotation report

The *Onchidella celtica* genome assembly (GCA_963931925.1) was annotated by Ensembl at the European Bioinformatics Institute (EBI), using the Ensembl genebuild for non-vertebrate species pipeline. This annotation includes 22 534 transcribed mRNAs from 12 582 protein-coding and 3 508 non-coding genes. The average transcript length is 14 864.72 bp, with an average of 1.40 coding transcripts per gene and 7.00 exons per transcript. For further information about the annotation, please refer to the annotation page on Ensembl.

### Wellcome Sanger Institute – Legal and Governance

The materials that have contributed to this genome note have been supplied by a Darwin Tree of Life Partner. The submission of materials by a Darwin Tree of Life Partner is subject to the **‘Darwin Tree of Life Project Sampling Code of Practice’**, which can be found in full on the Darwin Tree of Life website. By agreeing with and signing up to the Sampling Code of Practice, the Darwin Tree of Life Partner agrees they will meet the legal and ethical requirements and standards set out within this document in respect of all samples acquired for, and supplied to, the Darwin Tree of Life Project. Further, the Wellcome Sanger Institute employs a process whereby due diligence is carried out proportionate to the nature of the materials themselves, and the circumstances under which they have been/are to be collected and provided for use. The purpose of this is to address and mitigate any potential legal and/or ethical implications of receipt and use of the materials as part of the research project, and to ensure that in doing so we align with best practice wherever possible. The overarching areas of consideration are:

- Ethical review of provenance and sourcing of the material
- Legality of collection, transfer and use (national and international)

Each transfer of samples is further undertaken according to a Research Collaboration Agreement or Material Transfer Agreement entered into by the Darwin Tree of Life Partner, Genome Research Limited (operating as the Wellcome Sanger Institute), and in some circumstances, other Darwin Tree of Life collaborators.

## Data Availability

European Nucleotide Archive: Onchidella celtica (Celtic sea slug). Accession number PRJEB71538. The genome sequence is released openly for reuse. The *Onchidella celtica* genome sequencing initiative is part of the Darwin Tree of Life Project (PRJEB40665) and the Sanger Institute Tree of Life Programme (PRJEB43745). All raw sequence data and the assembly have been deposited in INSDC databases. The genome will be annotated using available RNA-Seq data and presented through the Ensembl pipeline at the European Bioinformatics Institute. Raw data and assembly accession identifiers are reported in Table 1 and Table 2. Production code used in genome assembly at the WSI Tree of Life is available at https://github.com/sanger-tol. Table 5 lists software versions used in this study.

## Author information

Contributors are listed at the following links:

- Members of the Marine Biological Association Genome Acquisition Lab
- Members of the Darwin Tree of Life Barcoding collective
- Members of the Wellcome Sanger Institute Tree of Life Management, Samples and Laboratory team
- Members of Wellcome Sanger Institute Scientific Operations – Sequencing Operations
- Members of the Wellcome Sanger Institute Tree of Life Core Informatics team
- Members of the Tree of Life Core Informatics collective
- Members of the Darwin Tree of Life Consortium
