# Supplementary material for: The genome sequence of the Celtic sea slug, Onchidella celtica (Audouin & Milne-Edwards, 1832) (Systellommatophora: Onchidiidae)
Source: Wellcome Open Res. 2025 Oct 6;10:557. [Version 1] doi: 10.12688/wellcomeopenres.25000.1 (PMC12744200; doi:10.12688/wellcomeopenres.25000.1)
Supplement: Peer review history

# Reviewer report: Reviewer response for version 1

**DOI:** 10.21956/wellcomeopenres.27538.r140727

**Published:** 2025-12-27

**Authors:** Erich M. Schwarz

**Affiliations:** 1 Cornell University, Ithaca, New York, USA

This data note describes the first chromosomal-quality genome assembly for Onchidella celtica, with a useful set of gene predictions by Ensembl. The general process of genome assembly, curation, and annotation is precisely described using methods that are state-of-the-art. This text has had a previous review by Dr. Sujan Kumar Datta that raised reasonable points, and it would appear that this version is intended to address those points.

 1. It would appear that Figure 5 is meant to address Dr. Datta's question about contamination or non-target sequences. However, I cannot see any discussion of the biological interpretation or practical response to Figure 5's data in the text; there is a section of Methods describing how Figure 5 was generated, but nothing I can find about what it means or what was done about it. Could the authors please add these?

 2. No comparison of genome assembly traits to other gastropods or pulmonates was provided. However, this may not be necessary since the standard format of genome reports by WTSI's Tree of Life generally does not seek to provide such comparisons. Therefore, I believe this point can be ignored.

 3. Figures 2 and 3 now appear to me to be fully acceptable.

 4. The authors have added a pleasingly informative paragraph about the known biology of Onchidella celtica. More generally, the text reads well, with no obvious errors.

 In addition to the points raised by Dr. Datta, I see two other minor points that the authors may wish to address.

 5. In the Methods, the authors refer first to "a PowerMasher II tissue disruptor" (for nucleic acid extraction) and then to "the Diagnocine Power Masher-II" (for Hi-C). Unless these really were two different pieces of laboratory equipment, could the authors please pick a single set of words to describe this piece of machinery and use it in both places?

 6a. In the title and elsewhere in the text, the authors describe this species as "Onchidella celtica (Audouin & Milne-Edwards, 1832)". However, I noticed that among the references they cite, this species went several different taxonomic designations: "Onchidella celtica (Cutler, 1817)" [Barfield, 2003]; "Onchidella celtica (Forbes and Hanley, 1852)" [Dron, 1971]; and "Onchidella celtica (Cuvier)" [Russell, 1925]. Which of these is correct? I believe the "(Audouin & Milne-Edwards, 1832)" designation is actually correct; could the authors cite a reference for it? Perhaps this would be a valid reference: Audouin, V.; Milne Edwards, H. (1832-1834). Recherches pour servir à l'histoire naturelle du littoral de la France. Paris: Crochard, 2 vols [vol. 1, 406 pp.; vol. 2, 290 pp., 8 pls].

 6b. Also, should this be written as "Audouin & Milne-Edwards, 1832" or "Audouin and Milne-Edwards, 1832"?

Are sufficient details of methods and materials provided to allow replication by others?

Yes

Is the rationale for creating the dataset(s) clearly described?

Yes

Are the datasets clearly presented in a useable and accessible format?

Yes

Are the protocols appropriate and is the work technically sound?

Yes

Reviewer Expertise:

Nematode genomics

I confirm that I have read this submission and believe that I have an appropriate level of expertise to confirm that it is of an acceptable scientific standard.

# Reviewer report: Reviewer response for version 1

**DOI:** 10.21956/wellcomeopenres.27538.r135438

**Published:** 2025-11-06

**Authors:** Dr. Sujan Kumar Datta

**Affiliations:** 1 Department of Zoology, University of Dhaka, Dhaka, Dhaka Division, Bangladesh

**Summary**

 This Data Note reports a high-quality, chromosome-level genome assembly and annotation of Onchidella celtica, the Celtic sea slug. Conducted within the framework of the Darwin Tree of Life (DToL) project, this work provides a valuable genomic resource for studies in molluscan systematics and evolution. It represents the first reference genome for the genus Onchidella, addressing a key gap in molluscan genomic resources. The assembly demonstrates excellent data quality (BUSCO 93.6%, QV 56, and 99.92% chromosomal assignment) and includes comprehensive documentation of protocols, software versions, and accession numbers. The manuscript is clearly written, methodologically rigorous, and adheres to the principles of open data and reproducibility.

**Areas for Improvement**

 1. The text could briefly mention whether any contamination or non-target sequences were identified and removed during curation using the ASCC pipeline, as this would strengthen confidence in the overall data quality.

 2. It would also be useful to include a short comparison of the genome size, GC content, or gene count relative to other gastropods or pulmonate species (e.g., *Onchidella floridana* or *Aplysia* species) to provide additional context and highlight the distinct features of this assembly.

 3. Figures: Figure 2 (k-mer plot)- label homozygous and heterozygous peaks for clarity. Include a short explanatory sentence in the legend of Figure 3 describing the main chromosomal pattern visible in the Hi-C map.

 4. Minor Textual Improvements: -Ensure consistent italicization of all species names.

 -Replace “megabases” with “Mb” throughout for consistency with other Tree of Life notes.

 -Consider a one-sentence summary of the ecological or conservation relevance of *O. celtica* (air-breathing, intertidal adaptation, patchy distribution).

 -In the second paragraph of the Genome Assembly section, an extra hyphen (“-”) has been inserted. Please remove it.

 - Remove the last citation (Blaxter et al., 2022) from the background section.

 - The article “The” can be omitted from the title as “Genome sequence of the Celtic sea slug, *Onchidella celtica* (Audouin & Milne-Edwards, 1832) (Systellommatophora: Onchidiidae).”

**Recommendation**

 This is a technically sound and well-documented genome report. With minor clarifications to figures and a short comparative context, it will make a valuable addition to the *Darwin Tree of Life* genome collection.

Are sufficient details of methods and materials provided to allow replication by others?

Yes

Is the rationale for creating the dataset(s) clearly described?

Yes

Are the datasets clearly presented in a useable and accessible format?

Yes

Are the protocols appropriate and is the work technically sound?

Yes

Reviewer Expertise:

Fisheries (Marine and Freshwater fishes, Crustacea, Mollusk), Aquaculture, Marine Biotechnology, Marine Products, Molecular Biology, Stock assessment

I confirm that I have read this submission and believe that I have an appropriate level of expertise to confirm that it is of an acceptable scientific standard.
